# Evaluation of humoral and cellular immune responses induced by a novel YidRv subunit vaccine from hypervirulent *Klebsiella pneumoniae*

**DOI:** 10.5114/bta/222175

**Published:** 2026-06-15

**Authors:** Tri Yudani M. Raras, Mauludy J. Ajrullah, Lailia N. Rahma, Ni Nyoman T. Puspaningsih, Rivo Y.B. Nugraha, Sumarno R. Prawiro

**Affiliations:** 1 Department of Biochemistry and Molecular Biology, Faculty of Medicine, Universitas Brawijaya, Malang, Indonesia; 2 Master Program in Biomedical Science, Faculty of Medicine, Universitas Brawijaya, Malang, Indonesia; 3 Department of Microbiology, Faculty of Medical and Health Science, Maulana Malik Ibrahim State Islamic University, Malang, Indonesia; 4 Laboratory of Proteomics, CoE Research Center for Biomolecule Engineering, Universitas Airlangga, Surabaya, Indonesia; 5 Department of Clinical Parasitology, Faculty of Medicine, Universitas Brawijaya, Malang, Indonesia; 6 Department of Clinical Microbiology, Faculty of Medicine, Universitas Brawijaya, Malang, Indonesia

**Keywords:** extended-spectrum β-lactamases, *Klebsiella pneumoniae*, yidRv, vaccine, immunogenicity

## Abstract

**Background:**

*Klebsiella pneumoniae* (KP) has re-emerged as a prominent etiological agent of pneumonia and systemic infection, with extended-spectrum β-lactamase (ESBL)-producing strains posing considerable therapeutic challenges. Moreover, vaccines may have limited efficacy because of the emergence of variant strains. The yidRv gene encodes a protein linked to excessive adherence and is conserved in over 99% of KP isolates. The present study aimed to explore the ability of a rec-YidRv protein to stimulate humoral and cellular immune response *in vitro* and *in vivo*.

**Materials and methods:**

The capability of the purified rec-YidRv protein to induce antibody synthesis was tested using a mice model. We also assessed whether rec-YidRv could successfully trigger the proliferation of splenocytes and the synthesis of their cytokines. Furthermore, the capacity of antigen to protect mice from ESBL-producing KP strain infection was investigated.

**Results:**

*In vitro* and *in vivo* analyses revealed that mice immunized with rec-YidRv generated a strong antibody and memory response. Compared to non-stimulated controls, immunized mice showed an increase in measurable KP-directed immunoglobulin G (IgG) levels, with elevated IgG1 and IgG2b fractions; additionally, splenocyte proliferation expanded by 5-fold. Infection of mice with a lethal dose of KP following immunization markedly extended animal survival and decreased the pathogen’s ability to form ventricular biofilms.

**Conclusions:**

The rec-YidRv elicited robust antibody responses and a moderate expansion of cellular activity. Rec-YidRv is an effective protein-only platform for inhibiting infection by antibiotic-resistant KP. Future studies should incorporate refined adjuvants and improved administration methods to enhance protective responses.

## Introduction

Extended-spectrum β-lactamase-producing *Klebsiella pneumoniae* (E-KP) strains are an emerging global health threat. E-KP has spread to 43 nations and exhibits multi-drug resistance, which has hindered therapy and increased mortality over the past decade (Ramatla et al. 2023). This strain is an opportunistic pathogen that routinely targets immunocompromised hosts; however, its ability to severely infect otherwise healthy cohorts is being increasingly recognized (Paczosa and Mecsas 2016). *Klebsiella pneumoniae* (KP) variants are currently harboring additional β-lactamase-encoding plasmids that confer resistance to the β-lactam backbone and to drugs that were previously effective, including sulfamethoxazole, aminoglycosides, trimethoprim, and fluoroquinolones (Ur Rahman et al. 2018). In the pursuit of novel therapeutic interventions, the modeling of a preventive, vaccine-mediated host defense has emerged as a rational and compelling alternative, grounded in historical success of immunological strategies aimed to mitigate bacterial diseases (Liang et al. 2022).

A new approach for protecting against pneumococcal disease is the development of protein-based subunit vaccines, which induces a rapid, robust immune response with minimal side effects (Ghattas et al. 2021). However, the application of this strategy to elicit immune responses against KP has been challenging because the subunits vary considerably among strains (Megías et al. 2018). To overcome this limitation, a bioinformatics scan identified the *yidR* gene, which encodes a putative ATP- and GTP-binding protein that enhances KP adherence and is virtually identical across almost all KP isolates. The sequence-based conservation of this protein renders recombinant YidR a promising target for vaccine development (Yang et al. 2019). Previous studies indicate that immunization with a DNA vaccine carrying *yidR* fused with interleukin 17 (IL-17) significantly enhances the adaptive immune response and provides superior protection against KP infection in vaccinated mice (Lv et al. 2024). The *yidRv* gene from hypervirulent KP (hvKP) isolated from pneumonia patients in Malang, Indonesia, exhibits 99.75% homology to the *yidR* gene (Permadi et al. 2024). The prevalence and sequence similarity suggest the feasibility of developing antigens against KP (Yang et al. 2019; Permadi et al. 2024); however, comprehensive studies of humoral and cellular immune responses to the YidRv antigen have not been conducted.

In our previous study, the *yidRv* gene was amplified from hvKP, cloned into the pET21a plasmid, and expressed in *Escherichia coli* (*E. coli*) BL21-DE3 (Raras et al. 2024). In the present study, we observed the potency of a novel recombinant YidRv (rec-YidRv) subunit protein as a pneumonia vaccine candidate through *in vitro* and *in vivo* analyses. The immunogenicity of the purified rec-YidRv was analyzed, with a focus on cellular and humoral immune responses. The antigen was also analyzed for its ability to protect mice from E-KP infection.

## Materials and methods

### Protein purification

The *yidRv* gene was cloned into the pET21a plasmid and transformed into *E. coli* BL21 (DE-3). The rec-YidRv was overexpressed as reported previously (Raras et al. 2024). Protein expression was induced with 1 mM isopropyl β-D-1-thiogalactopyranoside treatment for 16 h at 20°C. The cells were lysed by sonication in lysis buffer and centrifuged, and the supernatant was purified using a Ni-Sepharose HisTrap affinity chromatography column (Cytiva, Marlborough, USA) pre-equilibrated with binding buffer. The purity of rec-YidRv was assessed by SDS-PAGE (Laemmli 1970), and the proteins were stained with Coomassie Brilliant Blue.

### Mouse immunization protocol

**Figure 1:**
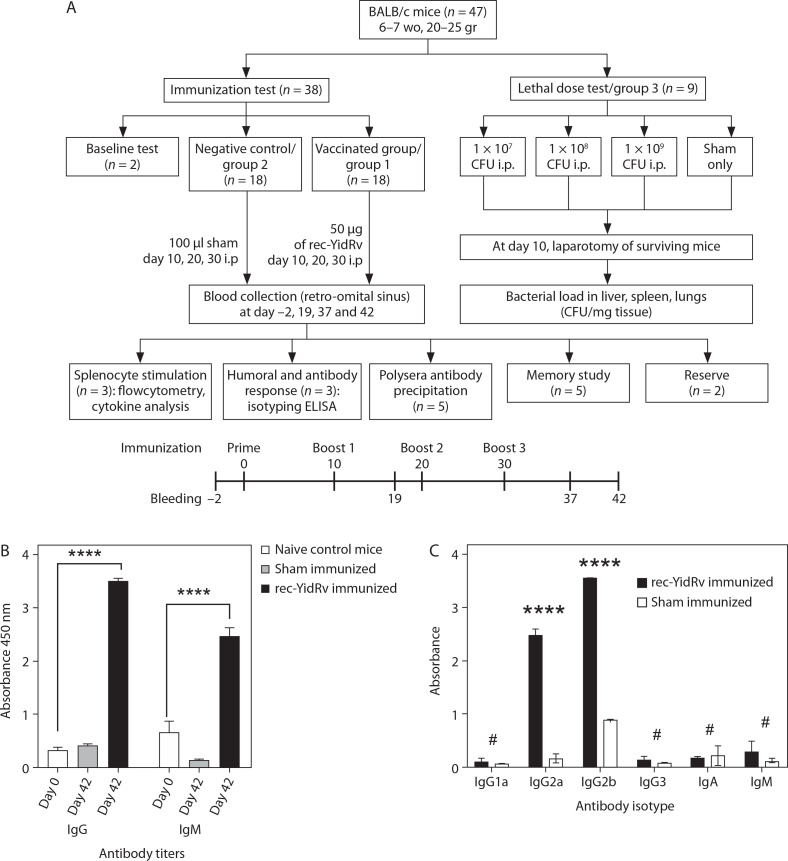
Antibody titers and isotype determination from recombinant YidRv (rec-YidRv)-induced anti-rec-YidRv serum in BALB/c mice. (**A**) Schematic representation of mice allocation, immunization, and blood collection schedules for the rec-YidRv antigen. (**B**) Estimation of the antibody titers of anti-rec-YidRv serum with IgG and IgM immunoglobulin production induced by 50 μg/ml rec-YidRv subunit vaccine at day-2 and 42. (**C**) Antibody isotypes induced by 10 μg/ml rec-YidRv were evaluated by ELISA using isotype-specific monoclonal antibodies for IgG1a, IgG2a, IgG2b, IgG3, IgA, and IgM. *p ≤ 0.05, **p ≤ 0.01, ***p ≤ 0.001, ****p ≤ 0.001, ^#^no significance

Female BALB/c inbred mice (*n* = 47, age: 6–7 weeks, average weight 20–25 g) were acclimatized under conventional laboratory conditions, with free access to food and water and a 12-h light-dark cycle for 1 week. Thirty-eight mice were used for the immunization test, and 9 mice for the lethal dose test. The animal procedures followed ethical guidelines and were approved by the Brawijaya Medical Faculty Animal Care and Use Committee (no. 104/EC/ KEPK/ 04/ 2024). On day-2 of vaccination, 2 mice from the immunization group were sacrificed for baseline evaluation. Following 1 week of acclimation, the remaining 36 mice were randomly allocated to 2 groups: rec-YidRv-immunized (treatment group, *n* = 18) and sham-immunized (negative control group, *n* = 18). Rec-YidRv-immunized mice and mice for the lethal dose test received 50 μg of the rec-YidRv protein mixed with phosphate-buffered saline (PBS) (pH 7.4) and 1.75 μg kanamycin in an emulsion containing Complete Freund Adjuvant (Sigma-Aldrich, USA) and Incomplete Freund Adjuvant (G-Bioscience, USA) in 1 : 1 ratio for the boosters (days 10, 20, and 30) (Raras et al. 2024). The injections were administered intraperitoneally (i.p.). Sham-immunized mice received 100 μl sham (PBS + adjuvant) i.p. at the same time intervals as rec-YidRv-immunized mice. To assess antibody response, blood samples were collected through the intracardiac blood collection on day-2 (baseline) and day-42. Sera were collected and stored at –80°C for antibody analysis. On day-42, three of the 18 immunized mice (rec-YidRv-immunized and Sham-immunized) were allocated for splenocyte stimulation experiments, humoral and antibody responses (*n* = 3), polysera antibody precipitation (*n* = 5), and memory study (*n* = 5), while the remaining mice served as reserves. In the lethal dose test group, all nine mice were assigned to the bacterial challenge. The mouse immunization protocol is presented in Figure 1A.

### Determination of serum antibody isotypes by ELISA

Serum antibody isotypes were determined using a Mouse Ig Isotyping ELISA kit (Mouse Ig Isotyping Uncoated ELISA Kit, Cat# 88-50630-86, Thermo Fisher Scientific, USA) in accordance with the manufacturer’s instructions. The assay was used to detect specific antibody isotypes, including immunoglobulins IgG1, IgG2a, IgG2b, IgM, and IgA. Briefly, serum samples obtained from immunized mice were serially diluted and added to ELISA plates coated with the corresponding capture antibodies. Serum from sham-immunized mice served as the negative control. After incubation and washing steps, the bound antibodies were detected using the appropriate enzyme-conjugated secondary antibodies provided in the kit. The optical density (OD) was measured at 450 nm using a microplate ELISA reader. The endpoint titer was defined as the highest serum dilution that produced an OD value greater than the mean OD of the negative control plus two standard deviations (SD).

### Splenocyte proliferation assay and cytokine analysis

Twelve days following the last booster (day-42), rec-YidRv-immunized mice (*n* = 3) and Sham-immunized mice (*n* = 3) were euthanized. The spleens were aseptically removed, homogenized, filtered through a 70-μm cell strainer, and treated with red blood cell lysis buffer (BioLegend, USA). The cells were washed and resuspended in complete Dulbecco’s Modified Eagle Medium (10% FBS, 1% penicillin/streptomycin) (Gibco, UK). The splenocytes were seeded onto 96-well plates in triplicate at the density of 1 × 10^6^ cells per well. The cells were then stimulated with three concentrations of rec-YidRv at 2, 4, and 10 μg/ml. Concanavalin A (10 μg/ml) (Sigma-Aldrich, Darmstadt, Germany) served as the positive control, and cell culture medium served as the negative control. The plates were incubated at 37°C in 5% CO_2_ for 72 h. Cell proliferation was measured using the WST-1 reagent (Roche, Germany). The Stimulation Index (SI) was calculated as follows:

SI = OD unstimulated/OD stimulated

The supernatants of the splenocyte culture were collected for cytokine analysis [interleukin-2 (IL-2), interferon-γ (IFN-γ), IL-17A, and tumor necrosis factor-α (TNF-α)] using ELISA kits (Elabscience, USA) in accordance with the manufacturer’s instructions. For the pellet containing splenocyte cells, by using fluorescence-conjugated antibodies, flow cytometry was used to assess the immune cell populations (CD3+, CD19+, CD11b+, and MHC II+) (BioLegend, Elabscience, USA). The analysis was performed using a BD FACSCalibur instrument (2–3 colors), and the data were processed using FlowJo software.

### In vitro antimicrobial susceptibility testing

To evaluate the neutralizing effects of antibodies, a 16 mg/ml antibody stock was diluted in PBS to obtain concentrations of 10, 20, 40, and 80 μg/ml, with a total volume of 100 μl. Additionally, 100 μl of hvKP suspension (10^7^ CFU/ml) was diluted in Mueller-Hinton (MH) broth (Sigma-Aldrich, USA) and added to each dilution. PBS and E-KP in MH broth without antibodies served as negative controls, and 50 μg/ml kanamycin served as the positive control. Absorbance measurements at OD_600_ were recorded before and after incubating the plates at 37°C overnight. The minimum inhibitory concentration (MIC) value for the polyclonal antibody was defined as the lowest dilution at which no increase in turbidity (OD) occurred after overnight incubation.

### Lethal dose test

To assess protection, mice were injected i.p. with different hvKP doses: Group A [1 × 10^7^ colony-forming units (CFU)/ml], Group B (1 × 10^8^ CFU/ml), Group C (1 × 10^9^ CFU/ml), and Control: sham only. On day 10, the surviving mice underwent laparotomy. Organs (liver, spleen, and lungs) were harvested, homogenized in PBS, and plated on Luria–Bertani agar to determine bacterial load (CFU/mg tissue).

#### Biofilm test

The effect of rec-YidRv antibodies on biofilm formation by E-KP was evaluated using a microtiter plate assay with minor adjustments (Chang et al. 2021). A mixture of 100 μl of 10^8^ CFU/ml bacterial suspension and 100 μl of varying concentrations of polyclonal antibody (12.5, 25, 50, and 100 μg/ml) was incubated in 96-well plates overnight at 37°C. E-KP without kanamycin and rec-YidRv antibodies, and E-KP with kanamycin were used as negative and positive controls, respectively. Following incubation, the plates were rinsed with PBS, and the biofilms were fixed with 99% methanol for 15 min. Crystal violet was added and dried. The biofilms were dissolved in 33% glacial acetic acid, and the OD was measured at 570 nm using a multimode plate reader (Tecan AG, Switzerland).

### Passive immunization and lethal bacterial challenge assay

The neutralization efficacy of the polysera was determined by administrating passive antibodies. Five-week-old naive female BALB/c mice (*n* = 5) were injected intraperitoneally with 200 μl of polysera, and after 24 h, they were challenged with 2 × LD100 (2 × 10^7^ CFU/ml) of E-KP. A control group (*n* = 3) was injected with the same quantity of PBS alone and challenged with the same quantity of lethal dose. On day 14, mice were examined to determine their survivability. Survival of mice post-challenge within 14 days was performed by Kaplan–Meier curves using GraphPad Prism 5.0 version.

### Statistical analysis

Data are presented as mean ± SD from two independent experiments performed in triplicate. The exact sample size (*n*) for each experiment is indicated in the corresponding figure legends. Graphical and statistical analyses were performed using GraphPad Prism version 10.2.3 (GraphPad Software Inc., CA, USA). Statistical differences between groups were evaluated using one-way ANOVA, followed by Tukey’s multiple comparison test. For repeated ELISA measurements, mixed-effects models were considered to address within-sample variability. Precise *p*-values are reported where applicable, and a *p*-value of < 0.05 was considered statistically significant.

## Results

### Purification of the YidRv protein, and SDS-PAGE

**Figure 2:**
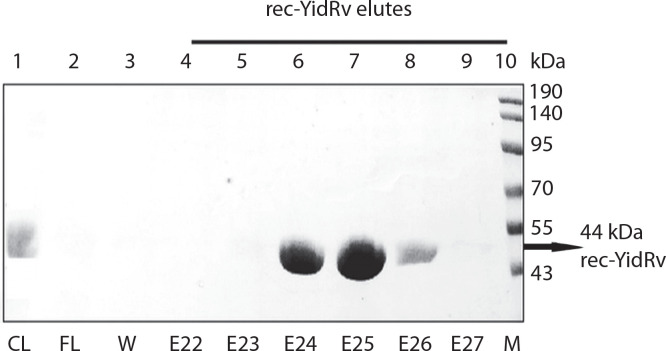
Purification of recombinant YidRv (rec-YidRv) using the immobilized metal affinity chromatography method. Sodium dodecyl sulfate–polyacrylamide gel electrophoresis analysis showing the rec-YidRv protein under different elution conditions appropriate to fast protein liquid chromatography purification. CL – crude lysate, E22–27 – elution fractions 22–27, FL – flowthrough, M – marker, W – washing

Sodium dodecyl sulfate–polyacrylamide gel electrophoresis (SDS-PAGE) analysis showed a distinct band in elution fractions 24, 25, and 26 following YidRv protein purification. The separated protein was validated by SDS-PAGE (Figure 2).

### Rec-YidRv immunization induces IgG subclass responses but not IgM production

Figure 1A summarizes the experimental flow, immunization types, immunization time points, and mouse allocation. Antibody titers against rec-YidRv were compared with PBS to assess IgG1 and IgG2 levels, which are indicative of Th2 and Th1 responses, respectively. KP-specific-specific IgG2 production was enhanced by rec-YidRv vaccination (Figure 1C). rec-YidRv drastically doubled IgG2b titers, although IgG2a titer increased moderately. None of the groups showed a significant increase in IgG, IgG1a, or IgG3 levels (Figure 1C), whereas rec-YidRv significantly generate IgM (Figure 1B).

### In vitro proliferation of splenocytes from rec-YidRv-immunized mice and cytokine analysis

**Figure 3:**
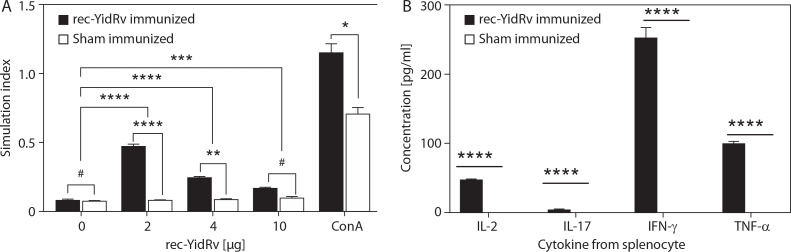
Proliferation of splenocytes isolated from BALB/c mice immunized with rec-YidRv and sham and treated with the rec-YidRv protein at different concentrations (2, 4, and 10 μg/ml) for 72 h (**A**). Cytokine analysis from recombinant (rec-YidRv)-immunized and sham-immunized mouse splenocytes. Bar graphs showing mean cytokine levels relative to positive controls in conditioned media of splenocytes isolated from rec-YidRv-immunized mice (**B**). Significance *p*-value summary examined by univariate ANOVA: *p ≤ 0.05, **p ≤ 0.01, ***p ≤ 0.001, ****p ≤ 0.001; ^#^no significance

The rec-YidRv antigen can induce the proliferation of splenocytes. This result was observed in the antigen-induced group, at the concentration of 2 μg/10^6^ cells; this concentration was sufficient to induce 5-fold higher proliferation compared to those without antigen. In contrast, splenocytes from the sham group showed no proliferation when induced without antigen (Figure 3A). Concanavalin A at 10 μg/well concentration was used as the positive control, and a remarkable increase in splenocyte proliferation was observed. This finding confirms successful cell culture process.

Single cells isolated from the spleens of the immunized group were stimulated with various concentrations of the rec-YidRv protein for 4 days to examine cytokine expression in splenocytes. Restimulation with rec-YidRv significantly enhanced IL-2-, IFN-γ-, and TNF-α-producing cell populations in the spleen immunized with rec-YidRv, but not IL-17A-producing cell population (Figure 3B). The sham mice group showed minimal induction of these cytokines.

### Flow cytometry analysis

**Figure 4:**
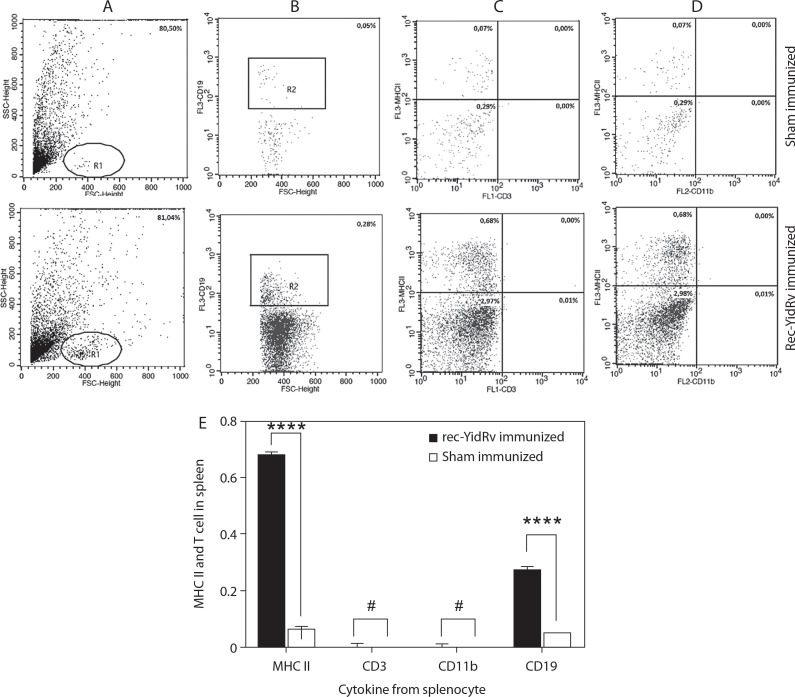
The percentage of MHC II and T cells from splenocytes was detected by flow cytometry after treatment with the rec-YidRv protein at different concentrations (2, 4, and 10 μg/ml) for 72 h (**E**) and sham treatment (**A–D**). (**A**) Scatter dot plot shows Region 1 (R1) exhibiting the lymphocyte population. (**B**) To explore the B lymphocyte population, gate R2 was created and marked with CD19. (**C**) The population of T cell lymphocyte was marked using CD3+ and analyzed within the LR (lower right) area; the MHC II population is shown within the UL (upper left) area. (**D**) Monocyte and dendritic cell population was analyzed from R1 marked with CD11b and counted within the LR quadrant. *p ≤ 0.05, **p ≤ 0.01, ***p ≤ 0.001, ****p ≤ 0.001, ^#^no significance

T cell responses were observed in the spleens of mice vaccinated with rec-YidRv and evaluated for the populations of immune T cells 12 days following the last immunization. MHC II and CD19 increased 3-fold in rec-YidRv protein-immunized mice (Figure 4). In contrast, following E-KP infection, CD3 and CD11b levels were decreased in the vaccination group and were not significantly different from those in the sham group.

### In vitro growth inhibition and in vivo protective efficacy of rec-YidRv antibodies against hvKP

**Figure 5:**
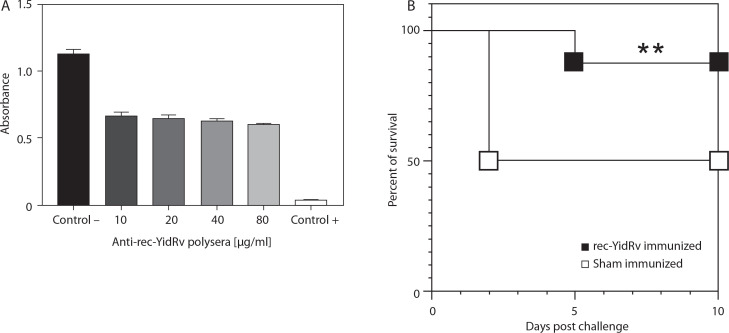
The minimum inhibitory concentration values of polyclonal antibodies from the anti-recombinant YidRv (anti-rec-YidRv) serum were in the range of 10–80 μg/ml. (**A**) Kaplan–Meier survival graph for rec-YidRv- and sham-immunized mice challenged with LD100 of extended-spectrum β-lactamase-producing *Klebsiella pneumoniae*. One animal succumbed to death (83% survival) in the active immunized group, whereas 50% of the animals survived in the sham-immunized group (**B**). Significance *p*-value summary: **p ≤ 0.01

The antibacterial activity of rec-YidRv antibodies against hvKP was evaluated using an *in vitro* growth inhibition assay. The MIC was defined as the lowest antibody concentration that can suppress bacterial proliferation. The MIC value ranged from 10 to 80 μg/ml. A marked reduction in bacterial growth was observed at 80 μg/ml, indicated by decreased absorbance values. This finding suggests the antibody exhibits substantial inhibitory activity against hvKP (Figure 5A). To further evaluate the *in vivo* protective efficacy of the antibody, a passive immunization model was generated and subjected to a lethal bacterial challenge. Survival analysis using Kaplan-Meier curves showed that rec-YidRv antibody-immunized mice had a higher survival rate compared to control mice. Specifically, 83% of antibody-treated mice survived the hvKP challenge, whereas only 50% of control mice survived following hvKP infection (Figure 5B). Statistical analysis revealed a significant difference in survival between the two groups on day 10 post-infection (*p* ≤ 0.01). Collectively, these findings show that rec-YidRv antibody immunization confers protective effects against hvKP infection by inhibiting bacterial growth *in vitro* and improving host survival *in vivo*.

### Biofilm inhibition

**Figure 6:**
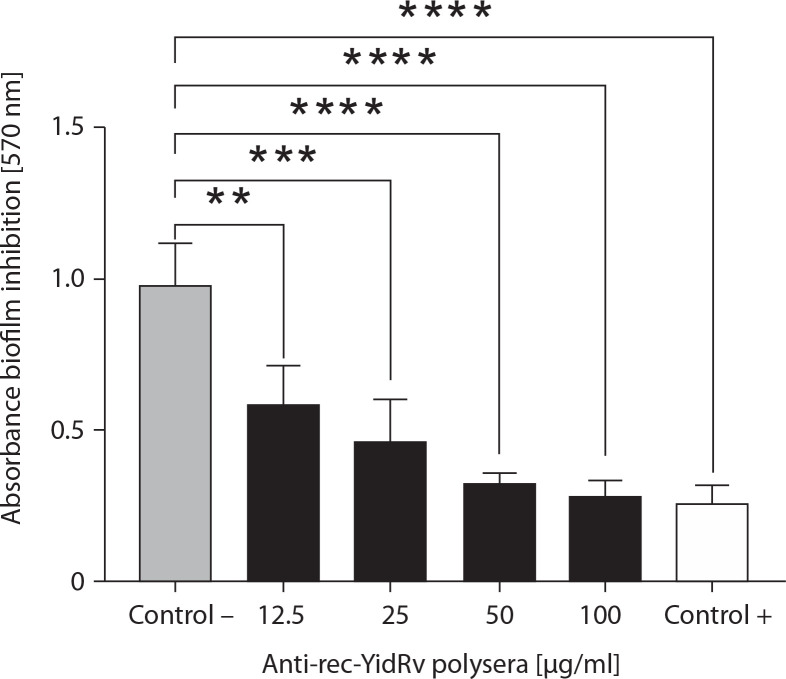
Inhibition of biofilm formation using different concentrations (12.5, 25, 50, and 100 μg/ml) of recombinant YidRv (rec-YidRv) antibodies. Percent reduction of biofilm formation by extended-spectrum β-lactamase-producing *Klebsiella pneumoniae* dose-dependently aligned with the concentration of anti-rec-YidRv polysera

Figure 6 shows the biofilm inhibitory activity of anti-rec-YidRv antibodies against KP. It depicts the inhibition percentages at various concentrations of anti-YidRv antibodies (12.5, 25, 50, and 100 μg/ml). The inhibition percentage correlated positively with an increase in YidRv antibody concentrations. The highest concentration (100 μg/ml) induced significantly greater biofilm inhibition, suggesting a direct relationship between antibody concentration and biofilm disruption efficacy.

## Discussion

E-KP continues to pose significant challenges as a prevalent source of severe pneumonia and systemic illnesses in pediatric and geriatric populations (Chang et al. 2021). Although subunit vaccines targeting common KP serotypes are available, the overall incidence remains high and is driven partially by extensive strain heterogeneity (Chang et al. 2021). Therefore, an effective vaccine must achieve broad serotype coverage and induce long-lasting, high-quality protective immunity.

Our study focused on the highly conserved *yidRv* gene with 99.75% sequence homology to the chromosomal *yidR* gene in numerous KP isolates (Yang et al. 2019). Because of its association with hyper-adhering traits, YidRv has emerged as an ideal target for subunit vaccine formulation. Immunization with the rec-YidRv protein induced vigorous humoral immunity and provided measurable *in vivo* protection against E-KP challenge. The observed immunoprotective effects are consistent with other studies reporting the attenuation of disease severity by YidR in multiple infection models, including bovine mastitis and murine pneumonia (Rodrigues et al. 2020).

The present study demonstrated that IgG2 production was enhanced by rec-YidRv vaccination. Immunization with rec-YidRv drastically doubled IgG2b titers, although IgG2a titer increased moderately. Humoral immunity is a key mediator of protection from infections, as peripheral IgG titers are significantly elevated post-vaccination, with a considerable shift toward the IgG2 subclass, indicating a Th2-oriented immunological profile (Babu et al. 2017). A higher IgG2 titer particularly against KP is a significant phenomenon because this IgG subclass improves opsonophagocytosis, enhances complement deposition, and promotes the clearance of encapsulated pathogens, which reduces systemic bacterial dissemination and infection severity (Sebina and Pepper 2018).

Our experimental challenge revealed that 83% of immunized mice survived compared to only 50% of control mice; however, the values were lower compared to those obtained in previous studies on *YidR*-based vaccine (Rodrigues et al. 2020). This divergence in results might be due to the BALB/c strain selected in our laboratory, because it promotes the delivery of humoral antibodies through the Th2 pattern, whereas in earlier studies, the C57 heritage favored the production of Th1-type cellular immunity (Song and Hwang 2017). A related consideration is the heterogeneity of capsular elements that are discernible in hvKP compared to that in classical KP strains (Permadi et al. 2024).

YidRv vaccination also predicts adhesion suppression. The YidR family proteins contribute to the maturation of bacterial membrane enzymes and the assembly of the respiratory complexes (Klenner 2015; Seputra et al. 2021). In related bacterial models, such as *Enterococcus* and *Staphylococcus*, homologous coat polypeptides can self-assemble as amyloid polymers to reinforce biofilm strength (Whelan and Potts 2015; Taglialegna et al. 2020). Thus, in the present study, the antibodies induced in immunized mice capsulized biofilm *in vitro* in a graded dose-dependent manner. Similar responses have been observed for flagellin-based vaccine candidates against *Streptococcus mutans* (Sun et al. 2016).

Regarding the immune phenotype, vaccination primarily mobilized antigen-presenting and oprB-deficient cell subsets, as indicated by the altered levels of MHC class II and CD19 cells (Ten Broeke et al. 2013; Zhou et al. 2021). In contrast, the cumulative profile of CD3 remained stable, and cytotoxic T cells were moderately recruited (Fossati-Jimack et al. 2013). These results support the hypothesis that rec-YidRv primarily increases humoral immunity. However, cytokine analysis revealed a significant IFN-γ response, which may partially compensate for the low IL-17A levels. IFN-γ has a critical role in suppressing pulmonary KP infection (Moore et al. 2002; Moore and Siopes 2002; Happel et al. 2005; Lee et al. 2015); however, its effectiveness differs depending on the infection route (Moore et al. 2002). Thus, the balance of humoral and cellular immunity generated by rec-YidRv may explain the limited but considerable protection observed in our model.

Zheng (Lv et al. 2024) reported that a DNA vaccine carrying *YidR* fused with the IL-17 gene induced humoral and cellular immune responses. In contrast, rec-YidRv elicits more humoral immune responses compared to cellular immune responses. This phenomenon was evident by the increase in MHC II expression, followed by elevation of CD19 as an activation marker of B cells (Perez Vidakovics et al. 2010). High MHC II levels indicate strong activation of antigen-presenting cells in presenting antigens to T helper cells (Yuseff and Lennon-Dumenil 2013), which triggers an adaptive immune response. Thus, YidRv mostly exerts an effect on B-cell activation (Zhou et al. 2021), indicating that the immune response generated by the rec-YidRv protein predominantly involved antibody formation.

CD19 also enhances B cell sensitivity to induce opsonophagocytosis and bacterial lysis (Nilsson et al. 2011; Fossati-Jimack et al. 2013). This observation correlates with a significant increase in CD3+CD4 cell populations of splenocytes exclusively within the rec-YidRv vaccinated cohort. CD3+CD4+ is a marker for helper T cells, a subset of lymphocytes that express both receptors (essential for T cell activation). These cells coordinate the immune response by activating B cells, macrophages, and other immune cells through cytokines. An elevated level of the helper T cell population, together with high IFN-γ levels, could promote the switching to IgG2b class antibodies produced by B lymphocytes. The generated IgG2b antibodies can disrupt initial bacterial adhesion, induce bacterial agglutination, and disrupt YidRv protein function, thus inhibiting the initial organizational process necessary for biofilm maturation.

Immunization with rec-YidRv induces a strong systemic antibody response, and *in vitro* experiments revealed that the generated antibodies are protective in nature. Lee et al. (2015) demonstrated an essential role of T-helper lymphocytes in combating KP through cytokine production. The present study showed increased IFN-γ levels, potentially compensating for the reduced IL-17 level. Amezcua Vesely et al. (2019) stated that lung-resident CD4 TRM cells from the Th17 subset produce IFN-γ and release IL-4 to attenuate KP infection and hypersensitivity. IL-17A was not significantly increased in the spleens of vaccinated mice and failed to confer complete protection by day 10 against E-KP infection (Happel et al. 2005). This finding was consistent with the increase in IFN-γ observed post-immunization and infection. IFN-γ plays a critical role in controlling lung infection due to KP; however, YidRv administration does not confer systemic protection (Moore et al. 2002; Moore and Siopes 2002). The significance of IFN-γ in KP infection varied according to the infection route (Moore et al. 2002). IFN-γ is predominantly produced in the spleen, followed by a decrease in its serum concentration. In the present study, rec-YidRv elicited protective immunity against multidrug-resistant KP through complementary processes: potent IgG2-driven humoral responses and direct disruption of the biofilm. Although the current formulation of rec-YidRv conferred insufficient protection, the immune profile warrants the development of YidRv as a subunit vaccine candidate. The contribution of YidRv to the actual protection of the host against KP infection should also be determined. The present study reveals the dual efficacy of rec-YidRv antibodies in inhibiting bacterial growth and enhancing host survival during infection. The results substantially contribute to establish YidRv as a valuable antigen and demonstrate the capabilities of modern vaccine-based platforms to simplify the development and delivery of effective vaccines against multidrug-resistant bacteria.

The present study has several limitations. Although YidRv immunization can enhance humoral responses and improve the mouse survival rate, a partial protective effect is induced. Consequently, vaccines combining several antigenic peptides are required to generate antibodies against various KP virulence factors. Moreover, despite an increase in the levels of CD3^+^, CD4^+^, and IFN-γ as Th1 activation markers, mucosal effector activity was not defined in this study. KP infection, accompanied by biofilm formation, typically begins on mucosal surfaces and medical devices. Therefore, future development is expected to focus on strengthening the mucosal Th17 response. Although the systemic infection model used in this study provided an overview of acute protection, its effect in clinical cases of nosocomial infection remains to be demonstrated. By utilizing adjuvant optimization, multi-antigen approaches, or next-generation vaccine platforms, YidRv appears to have potential to be incorporated as a part of a broader immunoprophylaxis strategy against resistant Gram-negative pathogens.

## Conclusions

rec-YidRv has quantifiable immunogenicity but provides limited protection against E-KP. Vaccinated mice showed strong IgG2-predominant humoral responses, durable memory, and a minimal amount of cellular T lymphocyte activation. Taken together, these immunological profiles yielded significant host survival and a contemporaneous decrease in the persistence of KP, which was supported by considerable biofilm inhibition. Although complete protection could not be rendered by the developed vaccine, the results substantiate YidRv as an attractive subunit vaccine candidate for further studies.
